# Clinical performance of a bulk-fill versus a nanofilled resin composite in non-carious cervical lesions with different extensions: a 6-years randomized, parallel, double-blind clinical trial

**DOI:** 10.1007/s00784-026-06778-y

**Published:** 2026-02-23

**Authors:** Ayla Macyelle de Oliveira Correia, Beatriz Coelho Marques, Luana dos Santos Souza, Eduardo Bresciani, Taciana Marco Ferraz Caneppele

**Affiliations:** 1 Department of Restorative Dentistry, Institute of Science and Technology, São Paulo State University – UNESP, São José dos Campos, São Paulo, Brazil; 2 Av. Francisco José Longo, 777, São José dos Campos, São Paulo, 12245-000 Brazil

**Keywords:** Composite resins, Bulk-fill, Incremental filling., Noncarious cervical lesions, Randomized controlled trial

## Abstract

**Objectives:**

To evaluate the clinical performance of a regular bulk-fill (Filtek Bulk Fill Posterior) and a regular nanofilled (Filtek Z350 XT) resin composite in restoring non-carious cervical lesions (NCCLs) with different occlusogingival distance (OGD).

**Methods:**

A randomized, parallel, double-blind controlled trial was conducted with 77 participants. One hundred and forty NCCLs were categorized by their OGD as 1.5 mm (±10%) or 3 mm (±10%). Lesions were randomized into four groups (n=35) and restored with Filtek Bulk Fill Posterior (1.5 mm-B and 3 mm-B) or Filtek Z350 XT (1.5 mm-C and 3 mm-C). The self-etch adhesive Clearfil SE Bond was used in all procedures. Two calibrated, blinded examiners evaluated the restorations at 6 years (range 66-94 months) using modified USPHS criteria. Survival analysis (retention/fracture) was performed using Kaplan-Meier (Log-rank test), and secondary outcomes were analyzed with Friedman’s and Wilcoxon tests (α=0.05).

**Results:**

After 6 years, 99 restorations were evaluated. Thirteen restorations were lost resulting in retention rates of 85.7% for 1.5 mm-C, 91.4% for 1.5 mm-B, 91.4% for 3 mm-C, and 91.4% for 3 mm-B. Significant marginal discrepancy was observed for 1.5 mm-B, 3 mm-C, and 3 mm-B (p < 0.01). Fifty-eight restorations showed minor marginal staining and 72 were rated Bravo for surface texture, with no significant differences among groups or compared to the 30-month evaluation (p>0.05). No significant difference was found for the other parameters.

**Conclusion:**

The regular bulk-fill demonstrated similar clinical performance to the regular nanofilled resin composite in NCCLs, regardless of the OGD after 6 years of clinical service.

**Clinical significance:**

This is the first 6 years clinical trial showing the long-term behavior of a bulk-fill resin composite in NCCLs. The OGD of NCCLs appears not to influence the clinical performance of resin composite.

**Supplementary Information:**

The online version contains supplementary material available at 10.1007/s00784-026-06778-y.

## Introduction

Non-carious cervical lesions (NCCLs) correspond to a loss of tooth structure, usually involving enamel, dentin, and cementum, of non-bacterial origin [1, 2]. Their etiology is multifactorial and resulting from a combination of occlusal stress, friction (endogenous and exogenous mechanical wear), and biocorrosion mechanisms (chemical or electrochemical dental tissue degradation) [3–5]. Modifying factors such as saliva, diet, and general health issues should also be considered. The reported prevalence of NCCLs among adults is 46.7% with lesion severity generally increasing over time that confirming its importance and clinical relevance [4]. Age, gender, gastric disease, occlusal interferences, vigorous toothbrushing, and lifestyle habits such as smoking and alcohol consumption, significantly influenced the occurrence and progression of NCCLs [5–7]. Several clinical situations, including pain or sensitivity, possibility of pulpal exposure and compromised esthetics would warrant a restorative intervention [8, 9].

Restorative procedures of NCCLs are challenging. The largest part of the bonding substrate is dentin, besides in many of these lesions sclerotic dentin is present. This dentin exhibits structural differences, being heterogeneous, non-permeable due to the obliteration of dentinal tubules with sclerotic casts that prevent optimal resin tag formation, acid-resistant hypermineralized surface layer. These characteristics are potential obstacles for primer and resin infiltration that make the adhesive procedure even more difficult and prone to failure [5, 8, 10]. To maximize adhesion and clinical outcomes, various strategies have been employed, including different adhesion [11–13] and restorative strategies [14–17]. The use of bulk-fill resin composite for the restoration in NCCLs can offer a streamlined restoration technique, more compact fillings, and time savings, since thicker increments can be applied [18–20]. In addition, two characteristics of these materials, such as higher translucency that ensure proper curing at the bottom of thicker layers and reduction the polymerization shrinkage stress may appear advantageous for longevity of clinical protocols compared to the conventional materials [18, 21–23]. Despite promising results observed in laboratory settings [18–23] and short-term clinical studies regarding bulk-fill resin composite [14, 16], clinical recommendations should primarily rely on evidence gathered from long-term clinical studies.

Another factor which could contribute to premature failure of the resin composite restorations is extension of those lesions. Some studies showed that some cavity configurations might favor stress concentration at the bonded interface more than others [24–27]. These overall findings raising a concern about the performance of NCCLs of different extensions restored with resin composites.

Although many long-term clinical studies have been conducted to evaluate restorative strategies in NCCLs [11–13, 15, 28], long-term clinical evidence remains limited. Many previous clinical trials are characterized by heterogeneous lesion selection, relatively short follow-up periods, and the lack of stratification according to lesion dimensions, which may influence stress distribution and restoration behavior. Consequently, the impact of lesion extension on the long-term clinical performance of regular bulk-fill and regular nanofilled resin composites in NCCLs is still not well established. Therefore, this randomized clinical trial aimed to evaluate the 72-month clinical performance of regular bulk-fill and regular nanofilled resin composites in NCCLs with different occlusogingival distance.

## Methods and materials

### Ethics approval and protocol registration

The Institutional Review Board approved this study (72995517.4.0000.0077). This clinical study was registered in the Brazilian Clinical Trials Registry (ReBEC-www.ensaiosclinicos.gov.br) under the identification number RBR-6w5gwh and was conducted and reported in accordance with the Consolidated Standards of Reporting Trials statement (CONSORT) [29].

### Trial design, settings, and location of data collection

This was a randomized, controlled, parallel, double-blind (participants and examiners) clinical trial with four study groups and equal allocation ratio. The study was conducted at the clinic of the School of Dentistry. Participant recruitment occurred continuously between September 2016 and July 2018. All restorations were placed during this period by a single calibrated operator.

Due to the restrictions imposed by the COVID-19 pandemic, clinical recalls were temporarily interrupted, resulting in a broader follow-up interval for the 6 years evaluation (66–94 months).

### Sample size calculation

The sample size calculation utilized Sealed Envelope online software (Sealed Envelope Ltd. 2012. Power calculator for binary outcome equivalence trial. [Online] Available from: https://sealedenvelope.com/power/binary-equivalence/ [Accessed May 02 2016], relying on the reported the annual failure rate of 2.2% for two-step self-etch adhesives in NCCLs reported in a systematic review [30]. To detect a 15% difference among the tested groups, using an alpha of 0.05 and a power of 80%, a minimum sample size was 33 restorations per group. To account for potential dropouts, more than 5% of restorations were added, resulting in a final sample size of 35 restorations per group.

### Eligibility criteria

Two calibrated dental students examined a total of 128 participants and determined their eligibility according to the inclusion and exclusion criteria. Participants needed to be in good general and oral health. They should be at minimum 18 years old. They should have an acceptable oral hygiene level with a minimum of 20 teeth in occlusion, in these teeth, the presence of at least one NCCL to be restored that was deeper than 1 mm in vital canines or premolars without mobility, with an opposing and adjacent tooth, and have an OGD of 1.5–3 mm (±10%).

Exclusion criteria encompassed participants with extremely poor oral hygiene severe, individuals suffering from chronic periodontal disease, those using orthodontic appliances, and individuals exhibiting severe bruxism. Smokers or participants undergoing tooth whitening procedures were excluded of the study.

Prior to the measurement of the OGD, a dental screening and dental prophylaxis (rubber cup, pumice, and water) were performed in all participants. Two-step silicone impressions (Express XT Putty Soft þ Express XT Light Body Quick; 3 M/ESPE, St Paul, MN, USA) were made of each tooth with NCCLs that met the inclusion criteria. To better visualize the NCCL margin, gingival retraction cords (Ultrapak #000 and Ultrapak #00; Ultradent Products, Inc, South Jordan, UT, USA) were used. The impressions were disinfected and poured with gypsum (Durone IV; Dentsply Sirona, York, PA, USA) 3 h after removal. The excess of material was removed from all surfaces, and then the cast was scanned with an extraoral scanner (inEos Blue; Dentsply Sirona, Vienna, Austria). The digitalized data were transmitted to a computer-aided design software program (Rhinoceros 4.0; McNeel North America, Seattle, WA. USA) in which the OGD (distance between the most apical point of the gingival margin to the occlusal margin, tracing a line parallel to the long axis of the tooth) of the lesions, was analyzed. Once this step was completed, the restorative treatment was randomly defined according to the generated sequence.

### Randomization and allocation concealment

The simple randomization process was performed using a website (www.random.org) by a staff member not involved in the research protocol with an equal allocation ratio for all comparison groups. Details of the allocated groups were recorded on cards contained in sequentially numbered, opaque, and sealed envelopes. To ensure the concealment of the random sequence and prevent selection bias, allocation assignments were revealed by opening the envelope immediately before the restorative procedure. The operator opened a sealed, opaque envelope containing the randomization details to determine the intervention according to the OGD determined previously (1.5–3 mm), using two resin options (nanofilled or bulk-fill). Randomization was performed at the restoration level. When a participant presented more than one eligible lesion, a predefined sequence was used to determine the order of allocation. In cases where lesions presented the same OGD, the tooth located in the quadrant with the lowest numerical order and positioned more mesial within that quadrant was assigned first. This standardized approach was applied to all participants to minimize allocation bias and ensure consistency in group assignment.

### Restorative procedures

The methods of restoration placement have already been published [16] and it is briefly described below. Before restorative procedures, the lesions were cleaned with pumice and water in a rubber cup to remove the salivary pellicle and any remaining dental plaque, followed by rinsing and drying. Following the American Dental Association guidelines (ADA) [31], the operators did not prepare any additional retention or bevel. The relative isolation method was performed in all procedures using gingival retraction cord (Ultrapak #000; Ultradent Products, Inc), cotton rolls, and a saliva aspirator.

The two-step self-etch adhesive Clearfil SE Bond (Kuraray America, Inc, New York, NY, USA) was used according to the manufacturer’s instructions. One coat of primer was applied on the entire lesion surface for 20 s, followed by gentle air for 5 s. Then, the adhesive was applied and light−cured for 10 s at 800 mW/cm^2^ (Radii cal, SDI, Victoria, Australia).

An experienced dentist with more than 5 years of dental practice experience restored all NCCLs. The composition of the restorative materials used are described in the previous publications [16, 32]. The resin composite Filtek Bulk Fill Posterior was used for half the lesions according to the predetermined OGD (1.5 mm ±10% and 3 mm±10%). This composite was applied in a single increment and light-cured for 40 s (800 mW/cm^2^, Radii cal, SDI, Victoria, Australia). Filtek Z350 XT was used in up to three increments for cavities with an OGD of 3 mm (± 10%) and in a single increment for cavities with an OGD of 1.5 mm (± 10%). Each increment was light-cured for 20s with an irradiance of 800 mW/cm^2^ (Radii cal, SDI, Victoria, Australia). The restorations were finished immediately with 12-fluted tungsten carbide burs (FG Bur; KG Sorensen, Barueri, SP, Brazil). Polishing was performed with a sequence of flexible disks (Soflex, 3 M ESPE, St. Paul, MN, USA) 7 days after placement. This delayed protocol was adopted to allow complete post-polymerization of the resin composite and stabilization of the adhesive interface, thereby reducing the potential influence of early surface manipulation on marginal integrity and surface characteristics.

### Calibration procedures

To ensure consistent and reliable assessments, two independent experienced dentists underwent a training process. For this, they observed 10 representative photographs of each score from each evaluation criteria. Also, these examiners assessed 10 patients, not involved in this study, were evaluated over the course of two consecutive days. Before the evaluations of the study participants, an intraexaminer and interexaminer agreement level of at least 85% [33].

### Blinding

The operator who performed the interventions was not blinded to the procedure. Nevertheless, the two examiners and all participants were blinded to the group allocation during all recalls.

### Clinical evaluation

All parameters during the evaluation were recorded using an individual standardized form by each evaluator at each recall time so that evaluators were kept blinded to earlier evaluations during the follow-up recalls. When disagreements occurred, a discussion led to a consensus. The evaluation of the restorations was performed at baseline (one week), and after 6, 12 [32], 24, 30 months [16], and 6 years (66–94 months) was conducted using.

by the modified United States Public Health Service (USPHS). Due to restrictions imposed by the COVID-19 pandemic, the 6-years recall could not be conducted at a single time point, resulting in a delayed and extended evaluation interval ranging from 66 to 94 months.

Retention/fracture was defined as the primary outcome, as it represents the most clinically relevant parameter for assessing restoration longevity and is widely adopted in NCCLs clinical trials. Other secondary outcomes were also evaluated: marginal staining, marginal adaptation, recurrence of caries, anatomic form, postoperative sensitivity, and surface texture. The postoperative sensitivity was evaluated one week after the restorative procedure, by applying an air stream from a dental syringe positioned 2 mm from the tooth surface and applied for 10s. These variables were ranked according to criteria Alpha, Bravo and Charlie.

### Statistical analysis

The statistical analysis followed the intention-to-treat protocol, according to CONSORT’s suggestion [29], which involved all teeth initially randomized, including those that were not evaluated at the specified time. These data related to dropout patients were replaced using the score of the last evaluation.

Because USPHS criteria generate ordinal data and normal distribution could not be assumed, nonparametric tests were applied. For the primary outcome fracture and retention, the survival rates of different groups were calculated by the Kaplan–Meier. Statistical analysis was performed individually using the Kruskal-Wallis test for each parameter (marginal staining, marginal adaptation, postoperative sensitivity, recurrence of caries) and considering each period of evaluation. The differences between the ratings of the four groups after 6 years were tested by Friedman’s repeated measures analysis of variance rank. Differences in the ratings of groups at each assessment period were compared using the Wilcoxon signed rank test. In all statistical tests, the significance level was preset at 5% (R statistical language R Studio, version 3.4.4, R Studio Team, Boston, MA, USA).

## Results

### Characteristics of included participants

**Fig. 1:**
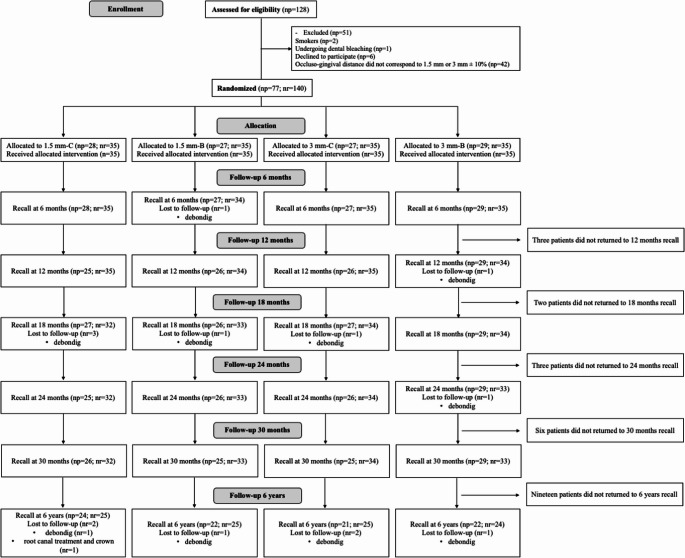
Flow diagram with details of study phases; 1.5 mm-C, NCCLs with OGD 1.5 mm restored with Filtek Z350 XT; 1.5 mm-B, NCCLs with OGD 1.5 mm restored with Filtek Bulk Fill Posterior; 3 mm-C, NCCLs with OGD 3 mm restored with Filtek Z350 XT; 3 mm-B, NCCLs with OGD 3 mm restored with Filtek Bulk Fill Posterior. NCCLs, noncarious cervical lesions; np, number of participants; nr, number of restorations; OGD, occlusogingival distance

Figure 1 shows the CONSORT flowchart detailing the flow of participants throughout the study. Of a total of 128 subjects assessed for eligibility, 51 were not enrolled because they did not fulfill the inclusion criteria. Thus, a total of 77 participants were enrolled in this study. One hundred and forty restorations were placed, 35 for each group. All details relative to the research subjects and characteristics of the restored cavities were described in the previous publications [16, 32].

Three participants did not attend to recall at the 12-month evaluation, 2 participants, to the 18-month recall, 3 participants to the 24-month recall, 6 to the 30-month recall. In this recall (66 until 94-month), 19 participants did not attend giving an overall recall rate of 75.3%. Of these 19 participants lost to follow-up, 2 patients passed away, 11 patients dropped out, 6 patients could not be contacted despite our efforts to reach them through their contact numbers, as well as their registered addresses.

The long-term evaluation originally planned for 6 years was completed over an extended interval (66–94 months) as a consequence of the COVID-19 pandemic. The mean follow-up time was similar among the groups: 1.5 mm-C (76.44 ± 5.6 months), 1.5 mm-B (76.04 ± 5.79 months), 3 mm-C (76.24 ± 5.75 months), and 3 mm-B (76.69 ± 5.57 months), with no statistically significant differences.

All data until 30-month have been described previously [16, 32]. Only the average follow-up period of 6 years data is described here.

### Retention/Fracture

**Fig. 2:**
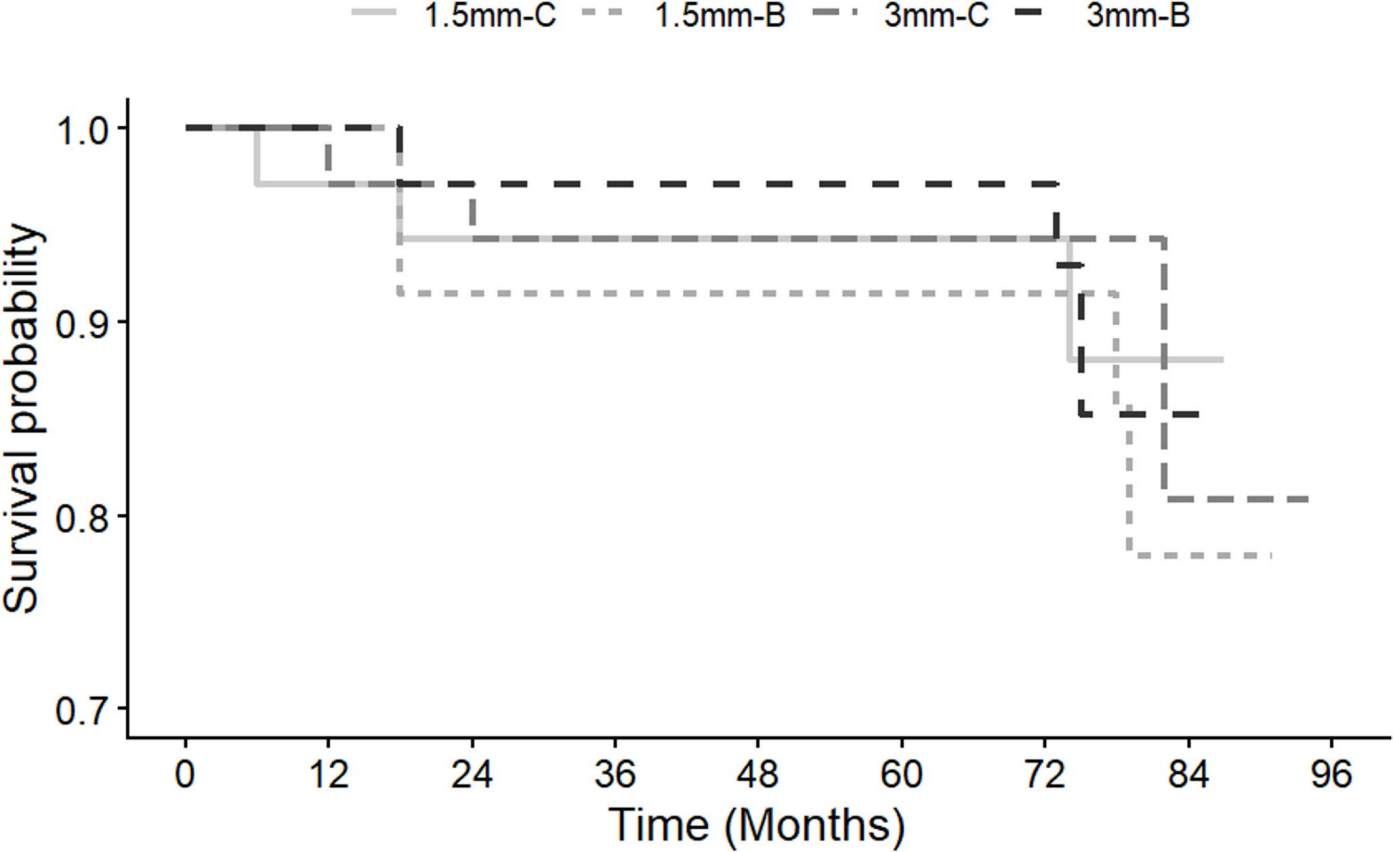
Survival curves for all groups (1.5 mm-C, NCCLs with OGD 1.5 mm restored with Filtek Z350 XT; 1.5 mm-B, NCCLs with OGD 1.5 mm restored with Filtek Bulk Fill Posterior; 3 mm-C, NCCLs with OGD 3 mm restored with Filtek Z350 XT; 3 mm-B, NCCLs with OGD 3 mm restored with Filtek Bulk Fill Posterior. NCCLs, noncarious cervical lesions; OGD, occlusogingival distance)

The survival rate of the restorations at the 6-year recall was 90,71%, considering as failures charlie scores for retention and fracture criteria. Thirteen restorations were lost after 6 years of clinical evaluation (5 for 1.5 mm-C; 3 for 1.5 mm-B; 3 for 3 mm-C; and 3 for 3 mm-B). The 6 years retention/fracture rates (95% CI) were 85.7% (74.1;97.3) for 1.5 mm-C; 91.4% (82.1;100) for 1.5 mm-B; 91.4% (82.1;100) for 3 mm-C; and 91.4% (82.1;100) for 3 mm-B. The Kaplan-Meier curves did not show any significant differences (Log-rank test, *p* = 0.961) among the cumulative probability of fracture and retention (Fig. 2).

### Marginal adaptation

Based on the modified USPHS criteria, after this recall (66 until 94-month), 19 of the restorations (13.57%) were scored as bravo (3 for 1.5 mm-C, 4 for 1.5 mm-B, 6 for 3 mm-C, and 6 for 3 mm-B) showed some marginal discrepancy. The 1.5 mm-B, 3 mm-C and 3 mm-B groups shows an increase in marginal discoloration over time (*p* < 0.01), while 1.5 mm-C group maintained the performance observed over 30 months of follow-up.

### Marginal staining

Fifty-eight restorations (41.42%) at the 72-month follow-up showed minor marginal staining (9 for 1.5 mm-C, 14 for 1.5 mm-B, 19 for 3 mm-C, and 16 for 3 mm-B).

When comparing the alpha to bravo scores over time (30-month data vs. the present recall), minor marginal staining was observed in 3 restorations of 1.5 mm-B group, 1 for 3 mm-C, 2 for 3 mm-B. No statistically significant differences were detected among the groups over the follow-up period (*p* > 0.01).

### Surface texture

Seventy-two restorations at the 6-year follow-up were scored as Bravo for surface texture (17 for 1.5 mm-C, 17 for 1.5 mm-B, 16 for 3 mm-C, and 22 for 3 mm-B). No significant differences were found among the groups at this recall (*p* > 0.01). When comparing the 30-month and 6 years evaluation results, no significant difference was detected (*p* > 0.01).

### Other parameters

No restoration showed evidence of recurrence of caries, postoperative sensitivity, and alteration of anatomic form Thus, they rated as alpha according to the modified USPHS criteria, after 6-year follow-up.

## Discussion

In the present study, restorations placed using the regular bulk-fill resin composite showed similar performance to those placed with the regular nanofilled resin composite, regardless of the OGD, after 6 years of clinical evaluation. This makes this study one of the most comprehensive evaluations to date. The retention rate observed was 85.7% for 1.5 mm-C and 91.4% for other groups. When the same two-step self-etch adhesives were applied in NCCL, after 24-months of clinical evaluation, the annual failure rate was 2.2% [30]. Distinctive results were found for this same follow-up period, Clearfil SE Bond showed 100% retention rates in NCCLs restorations [34]. Adhesives with a mild pH, such as Clearfil SE Bond, enhance interaction with dental tissues. The functional monomer as 10-methacryloyloxydecyl dihydrogen phosphate (10-MDP) presents in its composition enable the formation of a strong chemical bond with dental substrates and more stability in water than other monomers, even without additional mechanical retention, contributing to high retention rates [35]. It is important to emphasize that the high retention rate is also correlated to the favorable mechanical properties of the resin composites used. The regular bulk-fill has 76.5 wt% of inorganic fillers, whereas the regular nanofilled resin composite has 78.5 wt%.

In line with these findings, prior studies employing nanofilled resin composites and the same adhesive system (Clearfil SE Bond) for the restoration of NCCLs demonstrate a good clinical effectiveness like those reported in the present study in a short- and long-term follow-ups periods [34, 36–39]. Peumans et al. [36] reported a success rate of 86% after 13 years, while Van Meerbeek et al. [37] found 100% of restorations were successful after 2 years.

Considering these results, the possibility of working with bulk-fill resin composite may be simplify and expedite the restorative process in comparison to the layering technique, reducing chair time while addressing clinicians’ needs [40]. Studies showed up to 3.67 times faster when the restoration is performed with bulk-fill composite resin [41, 42]. Additionally, the bulk-fill placement technique reduces the number of voids and air bubbles, pulpal gaps, and contamination between increments, making the procedure less prone to technical errors [43]. From a clinical point of view, the bulk-fill resin composite has shown excellent clinical performance in different cavities with follow-ups ranging from 1 to 10 years [14, 16, 44–46].

At the 30-months of clinical evaluation, the marginal adaptation in all groups was statistically similar [16]. According to the manufacturer, the investigated regular bulk-fill resin composite presents monomers, such as an aromatic dimethacrylate (AUDMA) and additional fragmentation molecules (AFM), that allows the relaxation of the polymeric network, providing a potential stress relief and consequent decrease the polymerization shrinkage stress generated. Although this stress relief is related fewer marginal discrepancies and consequently decreases the risk of marginal staining compared to incremental resins [21], this pattern was not observed in this study during the period evaluated.

A randomized clinical study [34] tested self-etching adhesives containing 10-MDP or 2-hydroxyethyl methacrylate (HEMA) in its formulations and showed that Clearfil SE Bond showed the lowest marginal degradation at the 2-year follow-up. In this case, the stable chemical bonding of 10-MDP with the high mineral content of the enamel at the margin of the NCCLs may have contributed to the increase the durability of marginal adaptation. However, for at 6-years follow-up, the 1.5 mm-B, 3 mm-C and 3 mm-B groups presented statistical differences. Clearfil SE Bond has other relevant functional monomers in its composition, such as HEMA, which is highly hydrophilic [47–50]. This may have assisted to the degradation of the restoration margins over time, justifying the results observed.

Marginal staining was found in about 40% of the restorations after 6 years of clinical functioning, presenting statistical similarity with 30-months follow-up [16]. Marginal staining may occur due to the degradation of the adhesive systems, mainly for self-etching adhesives systems that contain HEMA or 10-MDP [34, 51]. Even after the adhesive polymerization, HEMA monomer will still exhibit water absorption properties due to the methacrylate groups that remain unreacted [52, 53]. In contrast, 10-MDP monomer forms strong bonds with calcium of the hydroxyapatite presents in enamel and dentin [35]. This interaction resulting in a salt, which do not dissolve easily and present high longevity, thus providing resistance and stability to the bonding interface even in an aqueous medium. The presence of both monomers in Clearfil Se Bond positively influence the clinical performance of the NCCLs restorations with respect to marginal staining. These marginal discrepancies are clinically acceptable, as they do not require further treatment and just can be addressed through refinishing and re-polishing of the restoration, indicating a very good clinical performance on NCCLs over time.

In this clinical trial, selective enamel etching was not performed. Some studies showed that this step did not have a significant influence on overall clinical performance of the restorations [36, 54–57], although there is a minor positive effect on marginal integrity and absence of marginal discoloration [36, 56, 57]. It is important also emphasized that these changes remain quite stable for medium-term clinical trials, until 5 years [36, 56, 57].

A previous studies showed that the occlusal forces and functional stress are different for type of tooth, thus influenced the longevity and clinical performance of restorative materials [1, 58, 59]. In this clinical trial, only premolars were included, as they are more likely to present NCCLs compared to anterior teeth, allowing a more standardized the evaluation of factors, restorative material and OGD.

Although lesion depth and overall geometry may influence stress distribution and restoration behavior, the present study focused exclusively on occlusogingival distance. Future studies incorporating three-dimensional lesion characteristics and finite element analyses may further elucidate their role in NCCL restoration longevity.

It is also worth noting that these results were observed for patients committed to their oral health, who voluntarily chose to participate and to receive oral check-ups at each follow-up. Given that various factors that can influence the longevity of composite restorations, including patient-related risk factors, especially those associated with lifestyle and health choices [60]. Thus, in a different scenario like limited access to healthcare service or low patient commitment to oral care, more degradation events could be observed, possibly within a shorter follow-up period.

A limitation of this study was the wide interval observed for the 6-year evaluation (66–94 months), mainly due to the COVID-19 pandemic. Although this heterogeneity may affect the uniformity of long-term assessments, the similar mean follow-up times among groups minimize the risk of systematic bias. Another limitation is that operator blinding was not possible due to the distinct handling characteristics and placement techniques required for the bulk-fill (single increment) versus the regular nanofilled resin composite (incremental technique). Considering the excellent results for the materials tested, it is not feasible to generalize these findings. While this follow-up results are promising, continued evaluation is necessary to fully understand the long-term clinical efficacy, durability of these restorations and the influence of the OGD in NCCLs.

## Conclusion

The bulk-fill resin composite maintained high survival and acceptable marginal integrity over six years in NCCLs with different OGD, demonstrated similar performance of a regular nanofilled resin composite.

## Supplementary Information

Below is the link to the electronic supplementary material.

Supplementary Material 1

Supplementary Material 2

## Data Availability

All data supporting the findings of this study are available within the paper and its Supplementary Information.

## References

[1] Aw TC, Lepe X, Johnson GH, Mancl LA (2002) Characteristics of noncarious cervical lesions: A clinical investigation. J Am Dent Assoc 133(6):725–733. 10.14219/jada.archive.2002.0268 12083648 10.14219/jada.archive.2002.0268

[2] Pecie R, Krejci I, Garcia-Godoy F, Bortolotto T (2011) Noncarious cervical lesions–a clinical concept based on the literature review. Part 1: prevention. Am J Dent 24(1):49–56 21469407

[3] Grippo JO, Simring M, Coleman TA (2012) Abfraction, abrasion, biocorrosion, and the enigma of noncarious cervical lesions: a 20-year perspective. J Esthet Restor Dent 24(1):10–23. 10.1111/j.1708-8240.2011.00487.x 22296690 10.1111/j.1708-8240.2011.00487.x

[4] Teixeira DNR, Thomas RZ, Soares PV, Cune MS, Gresnigt MMM, Slot DE (2020) Prevalence of noncarious cervical lesions among adults: a systematic review. J Dent 95:103285 10.1016/j.jdent.2020.103285 32006668 10.1016/j.jdent.2020.103285

[5] Goodacre CJ, Eugene Roberts W, Munoz CA (2023) Noncarious cervical lesions: morphology and progression, prevalence, etiology, pathophysiology, and clinical guidelines for restoration. J Prosthodont 32(2):e1–e18. 10.1111/jopr.13585 35920595 10.1111/jopr.13585

[6] Teixeira DNR, Zeola LF, Machado AC et al (2018) Relationship between noncarious cervical lesions, cervical dentin hypersensitivity, gingival recession, and associated risk factors: A cross-sectional study. J Dent 76:93–97. 10.1016/j.jdent.2018.06.017 29940290 10.1016/j.jdent.2018.06.017

[7] Maluf CV, Hirata R, Lourenço EJV et al (2025) Digital quantitative analysis of noncarious cervical lesions, occlusal tooth wear, and gingival recession: results from a 25-year clinical follow-up study. Clin Oral Investig 30(1):5. 10.1007/s00784-025-06686-7 41364373 10.1007/s00784-025-06686-7

[8] Tay FR, Pashley DH (2004) Resin bonding to cervical sclerotic dentin: A review. J Dent 32(3):173–196. 10.1016/j.jdent.2003.10.009 15001284 10.1016/j.jdent.2003.10.009

[9] Perez CR, Gonzalez MR, Prado NAS, de Miranda MSF, Macêdo MA, Fernandes BMP (2012) Restoration of noncarious cervical lesions: when, why, and how. Int J Dent 687058. 10.1155/2012/687058 10.1155/2012/687058 PMC3246729 22216032

[10] Hamdi K, Zaeneldin A, Samaha AH, Hamama HH (2025) Surface pretreatments for enhancing bond strength of resin composite to sclerotic dentin in non-carious cervical lesions: a systematic review. J Dent 163:106123. 10.1016/j.jdent.2025.106123 40987393 10.1016/j.jdent.2025.106123

[11] Tepe H, Celiksoz O, Yaman BC (2024) Clinical evaluation of single bond universal adhesive in non-carious cervical lesions: a 36-month retrospective study. Clin Oral Investig 29(1):33. 10.1007/s00784-024-06126-y 39731636 10.1007/s00784-024-06126-y

[12] Ñaupari-Villasante R, Carpio-Salvatierra B, Matos TP et al (2025) Six-year clinical evaluation of a copper-containing universal adhesive in non-carious cervical lesions: a split-mouth double-blind randomized clinical trial. J Dent 153:105532. 10.1016/j.jdent.2024.105532 39675690 10.1016/j.jdent.2024.105532

[13] Oz FD, Ozturk C, Soleimani R, Gurgan S (2022) Sixty-month follow up of three different universal adhesives used with a highly-filled flowable resin composite in the restoration of non-carious cervical lesion. Clin Oral Investig 26(8):5377–5387. 10.1007/s00784-022-04505-x 35477817 10.1007/s00784-022-04505-x PMC9045793

[14] Canali GD, Ignácio SA, Rached RN, Souza EM (2019) One-year clinical evaluation of bulk-fill flowable vs. regular nanofilled composite in non-carious cervical lesions. Clin Oral Investig 23:889–897. 10.1007/s00784-018-2509-8 29948275 10.1007/s00784-018-2509-8

[15] Kaida K, Kubo S, Egoshi T, Taira Y (2022) Eight-year clinical evaluation of two types of resin composite in non-carious cervical lesions. Clin Oral Investig 26(10):6327–6337. 10.1007/s00784-022-04587-7 35751704 10.1007/s00784-022-04587-7

[16] Correia AMO, Jurema ALB, Bresciani E, Caneppele TMF (2023) Effects of lesion size on the 30-month clinical performance of restorations with bulk fill and a regular nanofilled resin composite in noncarious cervical lesions. Clin Oral Investig 27(6):3083–3093. 10.1007/s00784-023-04914-6 36763143 10.1007/s00784-023-04914-6

[17] Favoreto MW, Condolo L, Carneiro TS, Wendlinger M et al (2024) Evaluation of preheating methods for bulk-fill thermoviscous composite in non-carious cervical lesions: a 24-month randomized controlled trial. J Dent 151:105409. 10.1016/j.jdent.2024.105409 39427958 10.1016/j.jdent.2024.105409

[18] Rosatto CM, Bicalho AA, Veríssimo C et al (2015) Mechanical properties, shrinkage stress, cuspal strain and fracture resistance of molars restored with bulk-fill composites and incremental filling technique. J Dent 43(12):1519–1528. 10.1016/j.jdent.2015.09.007 26449641 10.1016/j.jdent.2015.09.007

[19] Par M, Gamulin O, Marovic D, Klaric E, Tarle Z (2015) Raman spectroscopic assessment of degree of conversion of bulk-fill resin composites—changes at 24 hours post cure. Oper Dent 40(3):e92–e101. 10.2341/14-091-L 25275961 10.2341/14-091-L

[20] Chesterman J, Jowett A, Gallacher A, Nixon P (2017) Bulk-fill resin-based composite restorative materials: a review. Br Dent J 222(5):337–344. 10.1038/sj.bdj.2017.214 28281590 10.1038/sj.bdj.2017.214

[21] Rizzante FAP, Duque JA, Duarte MAH et al (2029) Polymerization shrinkage, microhardness and depth of cure of bulk fill resin composites. Dent Mater J 38(3):403–410. 10.4012/dmj.2018-063 10.4012/dmj.2018-063 30918231

[22] Correia AMO, Tribst JPM, Matos FS et al (2018) Polymerization shrinkage stresses in different restorative techniques for non-carious cervical lesions. J Dent 76:68–74. 10.1016/j.jdent.2018.06.010 29935253 10.1016/j.jdent.2018.06.010

[23] Correia A, Andrade MR, Tribst J, Borges A, Caneppele T (2020) Influence of bulk-fill restoration on polymerization shrinkage stress and marginal gap formation in class V restorations. Oper Dent 45:E207–E216. 10.2341/19-062-L 32243249 10.2341/19-062-L

[24] Correia AMO, Pereira VEM, Bresciani E, Platt JA et al (2019) Influence of cavosurface angle on the stress concentration and gaps formation in class V resin composite restorations. J Mech Behav Biomed Mater 97:272–277. 10.1016/j.jmbbm.2019.05.034 31136923 10.1016/j.jmbbm.2019.05.034

[25] Borges AL, Borges AB, Xavier TA, Bottino MC, Platt JA (2014) Impact of quantity of resin, C-factor, and geometry on resin composite polymerization shrinkage stress in class V restorations. Oper Dent 39(2):144–151. 10.2341/12-440-L 23786611 10.2341/12-440-L

[26] Braga RR, Koplin C, Yamamoto T, Tyler K, Ferracane JL, Swain MV (2013) Composite polymerization stress as a function of specimen configuration assessed by crack analysis and finite element analysis. Dent Mater 29(10):1026–1033. 10.1016/j.dental.2013.07.012 23937867 10.1016/j.dental.2013.07.012

[27] Han SH, Sadr A, Tagami J, Park SH (2016) Internal adaptation of resin composites at two configurations: influence of polymerization shrinkage and stress. Dent Mater 32(9):1085–1094. 10.1016/j.dental.2016.06.005 27372237 10.1016/j.dental.2016.06.005

[28] Omoto ÉM, Dos Santos PH, Shinohara MS et al (2025) Clinical performance of different adhesion strategies in non-carious cervical lesion restorations: a four-year randomized clinical trial. J Dent 153:105529. 10.1016/j.jdent.2024.105529 39674308 10.1016/j.jdent.2024.105529

[29] Schulz KF, Altman DG, Moher D, CONSORT Group (2011) CONSORT 2010 statement: updated guidelines for reporting parallel group randomised trials. Int J Surg 9(8):672–677. 10.1016/j.ijsu.2011.09.004 22019563 10.1016/j.ijsu.2011.09.004

[30] Peumans M, De Munck J, Mine A, Van Meerbeek B (2014) Clinical effectiveness of contemporary adhesives for the restoration of non-carious cervical lesions. A systematic review. Dent Mater 30(10):1089–1103. 10.1016/j.dental.2014.07.007 25091726 10.1016/j.dental.2014.07.007

[31] American Dental Association Council on Scientific Affairs (2011) Acceptance program guidelines: dentin & enamel adhesive materials. Am Dental Assoc 1–12. Available from: www.ada.org

[32] Correia A, Jurema A, Andrade MR, Borges A, Bresciani E, Caneppele T (2020) Clinical evaluation of noncarious cervical lesions of different extensions restored with bulk-fill or conventional resin composite: preliminary results of a randomized clinical trial. Oper Dent 45:E11–E20. 10.2341/18-256-C 31794342 10.2341/18-256-C

[33] Cvar JF, Ryge G (2005) Reprint of criteria for the clinical evaluation of dental restorative materials. 1971. Clin Oral Investig 9:215–232. 10.1007/s00784-005-0018-z 16315023 10.1007/s00784-005-0018-z

[34] de Oliveira RP, de Paula BLF, Alencar CM, Alves EB, Silva CM (2023) A randomized clinical study of the performance of self-etching adhesives containing HEMA and 10-MDP on non-carious cervical lesions: a 2-year follow-up study. J Dent 130:104407. 10.1016/j.jdent.2022.104407 36621551 10.1016/j.jdent.2022.104407

[35] Yoshida Y, Nagakane K, Fukuda R et al (2004) Comparative study on adhesive performance of functional monomers. J Dent Res 83(6):454–458. 10.1177/154405910408300604 15153451 10.1177/154405910408300604

[36] Peumans M, De Munck J, Van Landuyt K, Van Meerbeek B (2015) Thirteen-year randomized controlled clinical trial of a two-step self-etch adhesive in non-carious cervical lesions. Dent Mater 31(3):308–314. 10.1016/j.dental.2015.01.005 25637318 10.1016/j.dental.2015.01.005

[37] Van Meerbeek B, Kanumilli P, De Munck J et al (2005) A randomized controlled study evaluating the effectiveness of a two-step self-etch adhesive with and without selective phosphoric-acid etching of enamel. Dent Mater 21(4):375–383. 10.1016/j.dental.2004.05.008 15766585 10.1016/j.dental.2004.05.008

[38] Abdalla AI, El Sayed HY (2008) Clinical evaluation of a self-etch adhesive in non-carious cervical lesions. Am J Dent 21(5):327–330 19024260

[39] van Dijken JW (2010) A prospective 8-year evaluation of a mild two-step self-etching adhesive and a heavily filled two-step etch-and-rinse system in non-carious cervical lesions. Dent Mater 26(9):940–946. 10.1016/j.dental.2010.05.009 20646753 10.1016/j.dental.2010.05.009

[40] Tardem C, Albuquerque EG, Lopes LS et al (2019) Clinical time and postoperative sensitivity after use of bulk-fill (syringe and capsule) vs. incremental filling composites: a randomized clinical trial. Braz Oral Res 33(0):e089. 10.1590/1807-3107bor-2019.vol33.0089 31531552 10.1590/1807-3107bor-2019.vol33.0089

[41] Torres CR, Jurema AL, Souza MY, Di Nicoló R, Borges AB (2021) Bulk-fill versus layering pure Ormocer posterior restorations: a randomized split-mouth clinical trial. Am J Dent 34(3):143–149 34143584

[42] Torres CRG, Mailart MC, Moecke SE et al (2024) Flowable bulk-fill versus layering restorative material on class II restorations: a randomized clinical trial. J Dent 148:105154. 10.1016/j.jdent.2024.105154 38942111 10.1016/j.jdent.2024.105154

[43] Van Ende A, De Munck J, Lise DP, Van Meerbeek B (2017) Bulk-fill composites: a review of the current literature. J Adhes Dent 19(2):95–109. 10.3290/j.jad.a38141 28443833 10.3290/j.jad.a38141

[44] Yazici AR, Kutuk ZB, Ergin E, Karahan S, Antonson AS (2022) Six-year clinical evaluation of bulk-fill and nanofill resin composite restorations. Clin Oral Investig 26(1):417–426. 10.1007/s00784-021-04015-2 34110494 10.1007/s00784-021-04015-2

[45] Veloso SRM, Lemos CAA, de Moraes SLD et al (2019) Clinical performance of bulk-fill and conventional resin composite restorations in posterior teeth: a systematic review and meta-analysis. Clin Oral Investig 23(1):221–233. 10.1007/s00784-018-2429-7 29594349 10.1007/s00784-018-2429-7

[46] de Menezes AJO, do Nascimento Barbosa L, Leite JVC et al (2025) Clinical outcomes of bulk-fill resin composite restorations: a 10-year mapping review and evidence gap map. J Esthet Restor Dent 37(4):920–933. 10.1111/jerd.13339 39462873 10.1111/jerd.13339

[47] Nakabayashi N, Watanabe A, Gendusa NJ (1992) Dentin adhesion of modified 4-META/MMA-TBB resin: function of HEMA. Dent Mater 8(4):259–264. 10.1016/0109-5641(92)90096-u 1291394 10.1016/0109-5641(92)90096-u

[48] Burrow MF, Inokoshi S, Tagami J (1999) Water sorption of several bonding resins. Am J Dent 12(6):295–298 10850250

[49] Hitmi L, Bouter D, Degrange M (2002) Influence of drying and HEMA treatment on dentin wettability. Dent Mater 18(7):503–511. 10.1016/s0109-5641(01)00075-6 12191662 10.1016/s0109-5641(01)00075-6

[50] da Silva TSP, de Castro RF, Magno MB et al (2018) Do HEMA-free adhesive systems have better clinical performance than HEMA-containing systems in noncarious cervical lesions? A systematic review and meta-analysis. J Dent 74:1–14. 10.1016/j.jdent.2018.04.005 29649505 10.1016/j.jdent.2018.04.005

[51] Abdelkhalek E, Hamama HH, Mahmoud SH (2025) HEMA-free versus HEMA-containing adhesive systems: a systematic review. Syst Rev 14(1):17. 10.1186/s13643-025-02763-w 39838443 10.1186/s13643-025-02763-w PMC11748282

[52] Ferracane JL (1994) Elution of leachable components from composites. J Oral Rehabil 21(4):441–452. 10.1111/j.1365-2842.1994.tb01158.x 7965355 10.1111/j.1365-2842.1994.tb01158.x

[53] Michelsen VB, Moe G, Strøm MB, Jensen E, Lygre H (2008) Quantitative analysis of TEGDMA and HEMA eluted into saliva from two dental composites by use of GC/MS and tailor-made internal standards. Dent Mater 24(6):724–731. 10.1016/j.dental.2007.08.002 17889317 10.1016/j.dental.2007.08.002

[54] Perdigão J, Carmo AR, Anauate-Netto C et al (2005) Clinical performance of a self-etching adhesive at 18 months. Am J Dent 18(2):135–140 15973834

[55] Kubo S, Kawasaki K, Yokota H, Hayashi Y (2006) Five-year clinical evaluation of two adhesive systems in non-carious cervical lesions. J Dent 34(2):97–105. 10.1016/j.jdent.2005.04.003 15978714 10.1016/j.jdent.2005.04.003

[56] Can Say E, Özel E, Yurdagüven H, Soyman M (2014) Three-year clinical evaluation of a two-step self-etch adhesive with or without selective enamel etching in non-carious cervical sclerotic lesions. Clin Oral Investig 18(5):1427–1433. 10.1007/s00784-013-1123-z 24264636 10.1007/s00784-013-1123-z

[57] Perdigão J, Kose C, Mena-Serrano AP et al (2014) A new universal simplified adhesive: 18-month clinical evaluation. Oper Dent 39(2):113–127. 10.2341/13-045-C 23802645 10.2341/13-045-C

[58] Machado AC, Soares CJ, Reis BR, Bicalho AA, Raposo L, Soares PV (2017) Stress-strain analysis of premolars with non-carious cervical lesions: influence of restorative material, loading direction and mechanical fatigue. Oper Dent 42(3):253–265. 10.2341/14-195-L 28467256 10.2341/14-195-L

[59] Correia A, Bresciani E, Borges AB, Pereira DM, Maia LC, Caneppele T (2020) Do tooth- and cavity-related aspects of noncarious cervical lesions affect the retention of resin composite restorations in adults? A systematic review and meta-analysis. Oper Dent 45(3):E124–E140. 10.2341/19-091-L 32053461 10.2341/19-091-L

[60] Demarco FF, Cenci MS, Montagner AF et al (2023) Longevity of composite restorations is definitely not only about materials. Dent Mater 39(1):1–12. 10.1016/j.dental.2022.11.009 36494241 10.1016/j.dental.2022.11.009

