# Supplementary material for: Clinical performance of a bulk-fill versus a nanofilled resin composite in non-carious cervical lesions with different extensions: a 6-years randomized, parallel, double-blind clinical trial
Source: Clin Oral Investig. 2026 Feb 23;30(3):88. doi: 10.1007/s00784-026-06778-y (PMC12926243; doi:10.1007/s00784-026-06778-y)
Supplement: Supplementary file 1 — Supplementary Material 1 [file 784_2026_6778_MOESM1_ESM.docx]

Characteristics of the research participants, clinical characteristics, and distribution of NCCLs per group

| Characteristics | Number of participants (%) | | | | |
| --- | --- | --- | --- | --- | --- |
| Sex distribution |  | | | | |
| Male | 34 (44.1 %) | | | | |
| Female | 43 (55.8 %) | | | | |
| Age distribution (years) |  | | | | |
| 21-40 | 12 (15.6 %) | | | | |
| 41-60 | 52 (67.5 %) | | | | |
| 61-80 | 13 (16.9 %) | | | | |
| Characteristics of NCCLs | Number of lesions (%) | | | | |
|  | | 1.5 mm-C | 1.5 mm-B | 3 mm-C | 3 mm-B |
| Tooth distribution | |  |  |  |  |
| Canines | | 7 | 7 | 9 | 7 |
| Premolars | | 28 | 28 | 26 | 28 |
| Arch distribution | |  |  |  |  |
| Maxillary | | 15 | 25 | 16 | 14 |
| Mandibular | | 20 | 10 | 19 | 21 |
| Shape | |  |  |  |  |
| Saucer-shaped | | 15 | 15 | 23 | 24 |
| Wedge-shaped | | 16 | 17 | 7 | 3 |
| Mixed-shape | | 4 | 3 | 5 | 8 |
| Wear facets | |  |  |  |  |
| Yes | | 18 | 21 | 21 | 20 |
| No | | 17 | 14 | 14 | 15 |
| Preoperative sensitivity (air dry) | |  |  |  |  |
| Yes | | 18 | 22 | 20 | 15 |
| No | | 17 | 13 | 15 | 20 |

Abbreviations: 1.5 mm-C, NCCLs with OGD 1.5 mm restored with Filtek Z350 XT; 1.5 mm-B, NCCLs with OGD 1.5 mm restored with Filtek Bulk Fill; 3 mm-C, NCCLs with OGD 3 mm restored with Filtek Z350 XT; 3 mm-B, NCCLs with OGD 3 mm restored with Filtek Bulk Fill; NCCLs, noncarious cervical lesion; OGD, occlusogingival distance.
