# Supplementary material for: Clinical performance of a bulk-fill versus a nanofilled resin composite in non-carious cervical lesions with different extensions: a 6-years randomized, parallel, double-blind clinical trial
Source: Clin Oral Investig. 2026 Feb 23;30(3):88. doi: 10.1007/s00784-026-06778-y (PMC12926243; doi:10.1007/s00784-026-06778-y)
Supplement: Supplementary file 2 — Supplementary Material 2 [file 784_2026_6778_MOESM2_ESM.docx]

| **Evaluated criteria** | **Score** | **One week** | | | | **6 months** | | | | **12 months** | | | | **18 months** | | | | **24 months** | | | | **30 months** | | | | **6 years** | | | |
| --- | --- | --- | --- | --- | --- | --- | --- | --- | --- | --- | --- | --- | --- | --- | --- | --- | --- | --- | --- | --- | --- | --- | --- | --- | --- | --- | --- | --- | --- |
|  |  | **1.5 mm-C** | **1.5 mm-B** | **3 mm-C** | **3 mm-B** | **1.5mm-C** | **1.5mm-B** | **3 mm-C** | **3 mm-B** | **1.5mm-C** | **1.5mm-B** | **3 mm-C** | **3 mm-B** | **1.5mm-C** | **1.5mm-B** | **3 mm-C** | **3 mm-B** | **1.5mm-C** | **1.5mm-B** | **3 mm-C** | **3 mm-B** | **1.5mm-C** | **1.5mm-B** | **3 mm-C** | **3 mm-B** | **1.5mm-C** | **1.5mm-B** | **3 mm-C** | **3 mm-B** |
| Retention | Alfa | 35 | 35 | 35 | 35 | 35 | 34 | 35 | 35 | 33 | 33 | 34 | 34 | 32 | 33 | 34 | 34 | 32 | 33 | 34 | 33 | 32 | 33 | 34 | 33 | 30 | 32 | 32 | 32 |
|  | Charlie | -- | -- | -- | -- | -- | 1 | -- | -- | -- | 1 | -- | 1 | 3 | 2 | 1 | 1 | 3 | 2 | 1 | 2 | 3 | 2 | 1 | 2 | 5 | 3 | 3 | 3 |
| Marginal staining | Alfa | 35 | 35 | 35 | 35 | 33 | 32 | 33 | 35 | 29 | 29 | 25 | 31 | 26 | 26 | 22 | 27 | 25 | 24 | 20 | 24 | 23 | 22 | 16 | 19 | 21 | 18 | 13 | 16 |
|  | Bravo | -- | -- | -- | -- | 2 | 2 | 2 | -- | 4 | 4 | 9 | 3 | 6 | 7 | 12 | 7 | 7 | 9 | 14 | 9 | 9 | 11 | 18 | 14 | 9 | 14 | 19 | 16 |
| Marginal adaptation | Alfa | 35 | 35 | 35 | 35 | 34 | 34 | 35 | 35 | 32 | 33 | 34 | 33 | 31 | 33 | 34 | 33 | 31 | 33 | 34 | 31 | 29 | 33 | 32 | 29 | 27 | 28 | 26 | 26 |
|  | Bravo | -- | -- | -- | -- | 1 | -- | -- | -- | 1 | -- | -- | 1 | 1 | -- | -- | 1 | 1 | -- | -- | 2 | 3 | -- | 2 | 4 | 3 | 4 | 6 | 6 |
| Recurrence of caries | Alfa | 35 | 35 | 35 | 35 | 35 | 34 | 35 | 35 | 33 | 33 | 34 | 34 | 32 | 33 | 34 | 34 | 32 | 33 | 34 | 33 | 32 | 33 | 34 | 33 | 30 | 32 | 32 | 32 |
| Anatomic Form | Alfa | 35 | 35 | 35 | 35 | 35 | 34 | 35 | 35 | 33 | 33 | 34 | 34 | 32 | 33 | 34 | 34 | 32 | 33 | 34 | 33 | 32 | 33 | 34 | 33 | 30 | 32 | 32 | 32 |
| Postoperative sensitivity | Alfa | 32 | 32 | 32 | 32 | 32 | 33 | 32 | 35 | 33 | 31 | 32 | 32 | 32 | 32 | 34 | 34 | 31 | 33 | 32 | 32 | 32 | 33 | 34 | 33 | 30 | 32 | 32 | 31 |
|  | Bravo | 3 | 3 | 3 | -- | 3 | 1 | 3 | -- | -- | 2 | 2 | 2 | -- | 1 | -- | -- | 1 | -- | 2 | 1 | -- | -- | -- | -- | -- | -- | -- | 1 |
| Surface Texture | Alfa | 35 | 35 | 35 | 35 | 34 | 32 | 35 | 34 | 32 | 32 | 32 | 33 | 29 | 27 | 27 | 21 | 21 | 24 | 22 | 16 | 17 | 19 | 19 | 11 | 13 | 15 | 16 | 10 |
|  | Bravo | -- | -- | -- | -- | 1 | 2 | -- | 1 | 1 | 1 | 2 | 1 | 3 | 6 | 7 | 13 | 11 | 9 | 12 | 17 | 15 | 14 | 15 | 22 | 17 | 17 | 16 | 22 |

Number of analysed restorations for each group classified according to the Modified US Public Health Service (USPHS) criteria in different follow-up times.
